# Development and validation of the MARA scale in Spanish to assess knowledge and perceived risks and barriers relating to breast cancer prevention

**DOI:** 10.1007/s10552-021-01473-7

**Published:** 2021-07-08

**Authors:** Andrea Martínez-Urquijo, Álvaro Postigo, Marcelino Cuesta, María del Mar Fernández-Álvarez, Rubén Martín-Payo

**Affiliations:** 1 Hospital Cruz Roja Gijón, Gijón, Spain; 2 Facultad de Psicología, Universidad de Oviedo, Oviedo, Spain; 3 Facultad de Medicina y Ciencias de La Salud, Universidad de Oviedo, Oviedo, Spain; 4 Equipo de Investigación Precam, Instituto de Investigación Sanitaria del Principado de Asturias, Oviedo, Spain

**Keywords:** Breast neoplasm, Education, Health promotion, Female cancers, Patient education, Prevention

## Abstract

**Objective:**

The aim of this study was to develop a measurement instrument for assessing knowledge of breast cancer and perceived risk of developing the disease (MARA).

**Methods:**

641 women with a mean age of 36.19 years (SD = 7.49) participated in the study. Data collection took place during 2019 and included sociodemographic data, data on history of cancer and breast cancer, perceived risk, and feelings of concern about developing breast cancer. Internal consistency, test–retest reliability, convergent validity, and structural validity were tested.

**Results:**

The questionnaire items comprise 4 subscales: risk factors (9 items), signs and symptoms (9 items), perceived risk (6 items), barriers (7 items). A factor analysis revealed that the first two subscales had two dimensions each, whereas the other two subscales had one dimension each. Each subscale was shown to have adequate reliability (α = 0.74–0.92) and temporal stability (*r* = 0.201–0.906), as well as strong evidence of validity in relation to a questionnaire on breast cancer knowledge (*r* = 0.131–0.434). In addition, the subscales were shown to have high discriminatory power in terms of the presence or absence of a history of cancer or breast cancer, perceived risk, and feelings of concern.

**Conclusion:**

The MARA questionnaire represents a valid, reliable tool for assessing Spanish women’s knowledge, risks, perceptions, and barriers regarding breast cancer.

## Introduction

A large proportion of the most prevalent conditions of our time are associated with unhealthy behaviors. Specifically, there is evidence to suggest that engaging in healthy behaviors contributes to the prevention of cancer [1].

According to the health belief model (HBM), the following factors influence whether or not a person makes behavioral changes when faced with a potential risk of developing a condition: their perception of the severity of the condition, their susceptibility to the condition, the benefits that a change in behavior will have on prevention of the condition, and the perceived difficulties in implementing the recommended changes in behavior [2]. In addition, the amount of knowledge an individual possesses about the condition also influences their intentions to change their behavior. For instance, when an individual has adequate knowledge, they may have greater motivation and reflective capacity, which could positively influence their intention to change their behavior [3].

For these reasons, the availability of a tool for assessing an individual’s perceived risk, ability to engage in preventive behaviors, and knowledge about a particular condition is essential so that disease prevention and health promotion strategies can be implemented to encourage healthy lifestyles. This is even more important when it comes to breast cancer, which is one of the main health problems affecting women around the world, and Spain is no exception. A number of behaviors that can be addressed and modified play a major role in the development of breast cancer [4].

A recently published review explored the factors influencing women’s decisions to participate in breast cancer prevention programs, identifying the following: their perceived susceptibility to developing breast cancer, efforts required on their part to engage in healthy behaviors, their perceived competence, and their autonomy in carrying out the program, among others [5]. These findings reinforce the importance of women being aware of the signs and symptoms of breast cancer [6] and their ability to identify them early so that the interval between the onset of the first symptoms and the first consultation with their healthcare provider is as short as possible [4] and their prognosis can be improved [7]. In addition, identifying the risk of developing breast cancer would facilitate the development of interventions based on individual needs [4] coinciding with women’s preferences for receiving personalized information about their risks [5].

Several tools are currently available for assessing a woman’s level of knowledge of breast cancer and her perceived risk of developing the disease [8, 9]. These tools can be very helpful, as they allow women to identify their risks and enable health professionals to establish a personalized intervention in collaboration with each patient [10].

Being able to assess the knowledge of risk factors related to breast cancer, signs and symptoms of breast cancer, perceived individual risk of developing breast cancer, the ability to engage in preventive behaviors, and barriers to breast cancer prevention can help healthcare workers to meet women’s needs in relation to breast cancer prevention and create targeted prevention plans. This would also help women to become aware of their own health status.

### Aim

As we were unable to identify any validated questionnaires in Spanish specifically assessing a combination of the aforementioned aspects, the objective of our study is to design and analyze the metric properties of the MARA questionnaire to assess levels of knowledge of breast cancer, the perceived risk of developing it, and perceived barriers to engaging in preventive behaviors.

## Methods

### Participants

This validation study was carried out in 2019. Although there is no universal criterion, the usual recommendation is that each item should be answered by 5–10 individuals or include at least 200 observations (https://doi.org/10.7334/psicothema2016.209). A convenience sampling method was used. The eligibility criteria were (i) being at least the age of majority and (ii) not having been previously diagnosed with breast cancer.

Ethics approval was obtained from the Principality of Asturias Research Ethics Committee (protocol number 147/19). Women who were interested in participating were sent an informed consent form via email, which they then returned signed.

Women and trusted individuals who had collaborated with the research team on previous studies were contacted via email, text message, and social media. In addition, after 2–3 weeks, the instrument was administered again (test–retest) to a total of 30 women randomly selected from among the participants. At both points in time, in order to fulfill the aforementioned objectives, the participants received by email or text message a link to the self-administered questionnaire using Google Forms, to be completed online. Responses were automatically sent to an email address created specifically for this study and accessible only to members of the research team. Participant data were anonymized using randomized alphanumeric coding. No personal data that could directly identify participants were requested. The provisions of Spanish Organic Law 3/2018, of the 5th of December, on Personal Data Protection and Guarantee of Digital Rights were observed at all times [REF].

### Instruments

#### The MARA questionnaire

The MARA questionnaire was based on the b-CAS questionnaire [11], with the addition of eight questions: one question relating to individual aspects and seven relating to breast cancer knowledge. The b-CAS questionnaire consists of nine items on sociodemographic aspects and 53 items on different aspects of breast cancer divided into five categories: 1. knowledge of risk factors, 2. knowledge of signs and symptoms, 3. attitude toward breast cancer prevention, 4. barriers to early breast cancer screening techniques, and 5. displaying breast cancer prevention behaviors.

With the permission of Professor Rakkapao, the b-CAS questionnaire was translated into Spanish following the back-translation procedure proposed by the World Health Organization [12]. Initially, two native Spanish speakers with a good command of English translated the original version into Spanish independently. Both translations were independently compared by two researchers, who identified and amended any inaccurate or ambiguous expressions and concepts. Six items were removed, as their content was not suitable for the Spanish context. The consensus version was blind- and back-translated into English by an independent translator, whose rendition was then compared with the original by another member of the research team to ensure semantic equivalence. The questionnaire was administered to five women from outside the field of healthcare who assessed each item in order to test its understandability, acceptability, and applicability. The final version of the MARA questionnaire was then produced, comprising two sections. The first section contains 7 items on individual variables and the second section contains 47 items divided into five scales: risk factors (18 items), signs and symptoms (12 items), perceived risk and ability to engage in preventive behaviors (8 items), and barriers to engaging in preventive behaviors (9 items). Knowledge-related questions were coded with 3 response options (yes/no/I don’t know). Correct answers scored 1 point, while incorrect answers or no answer scored 0 points. Questions relating to “perceived risk of developing breast cancer” and “barriers to breast cancer prevention” were measured using a 5-point Likert scale ranging from 1 (strongly disagree) to 5 (strongly agree). In order to assess content validity, the MARA questionnaire was administered to 10 women, who assessed the clarity and understandability of its items. None of them reported any issues. The questionnaire was also sent to five experts in instrument development and healthcare (breast cancer care specialists), who assessed and amended the wording and position of the items, resulting in no items being removed or altered.

#### Knowledge, perceived risk, and concern regarding breast cancer

In the absence of a ‘gold standard’ instrument, the MARA questionnaire was administered along with questions written by Seven et al. [13]. The questionnaire consists of 12 questions measuring knowledge, perceived risk, and concerns regarding breast cancer. This instrument exhibited positive correlations between women’s perceived risk and level of concern, as well as with undergoing genetic testing [13]. The questionnaire response format is 0 = wrong answer/does not know and 1 = right answer. Reliability, as measured with Cronbach’s alpha, was 0.78. A positive, moderate relationship between the scores of the two questionnaires was hypothesized.

### Psychometric properties of the MARA

#### Descriptive statistics, reliability, and temporal stability of scores

The descriptive statistics of the items (mean, standard deviation, skewness, and kurtosis) were analyzed. The reliability of each scale was calculated using coefficient alpha for ordinal data [14] and McDonald’s coefficient omega [15]. In order to explore the stability of the scores, test–retest reliability was also calculated using intraclass correlation. A random-effects average measures model with absolute agreement was used to calculate intraclass correlation coefficients.

#### Validity

*Construct validity* was assessed using exploratory factor analysis (EFA). The unweighted least squares (ULS) method was used to estimate the parameters in the EFA, and the optimal implementation of parallel analysis (OIPA) was used to determine the number of dimensions of each scale [16]. In scales where the parallel analysis indicated more than one factor, oblique (oblimin) rotation was used. The comparative fix index (CFI) and the root mean square error of approximation (RMSEA) were used as adjustment statistics, with CFI > 0.95 and RMSEA < 0.08 indicating a good fit [17]. After studying the factor structures, Pearson’s correlations between the different scales of the questionnaire were analyzed. Evidence of validity in relation to other variables was tested using Pearson’s correlation between the MARA and a breast cancer knowledge questionnaire by Seven et al. [13]. Evidence of validity with regard to discriminatory power was studied by analysing possible differences between scales in terms of the presence or absence of a family history of cancer and breast cancer. To this end, a *t*-test for independent samples was conducted by comparing means. An ANOVA was performed to check for any differences in perceived risk (none/low/moderate/high) and feelings of concern about developing breast cancer (none/low/moderate/high). To identify groups between which there were differences, a Bonferroni post hoc test was carried out. *T* tests for independent samples were also performed based on the sociodemographic variable ‘level of education’ (having or not having a university education) on the subscales of the MARA instrument. Effect sizes were also calculated via Cohen’s *d*, with 0.2–0.4 indicating a small effect size, 0.5–0.7 indicating a moderate effect size, and 0.7 and above indicating a large effect size [18].

The various EFAs and reliability tests were performed using the program FACTOR 10.5.03 [19]. The other analyses were carried out using SPSS 24 (IMB Corp, 2016).

## Results

### Characteristics of the participants

A total of 641 women, all Spanish nationals, with a mean age of 36.19 years (SD = 7.49) participated. 72.4% of them had a university education. 48.8% were single, 44.9% were married, 6.1% were divorced, and 0.2% were widowed. 77.4% had a history of cancer, and 28.1% had a history of breast cancer. The subsample of women who took the retest had a mean age of 31.17 years (SD = 6.15). 60% of them were single, 26.7% were married, and 13.3% were widowed or separated. 60% had a history of cancer and 23.3% had a history of breast cancer. 90% had a university education.

### Psychometric properties

#### Construct validity

Items with factor weights below 0.30 were eliminated. As a result, 9 items on the risk factors scale, three items on the signs and symptoms scale, two items on the perceived risk scale, and two items on the barriers scale were eliminated. Table 1 shows the final number of items (31). The OIPA suggested two dimensions for the risk factors and signs and symptoms scales, and a single dimension for the perceived risks and barriers scales. Model fits were adequate for all scales (Table 1). Table 2 shows the factorial weights for each item, all of which exceed 0.45. The correlations between the two dimensions that make up the risk factors scale and the signs and symptoms scale are adequate (*r* = 0.40 and *r* = 0.30, respectively).

**Table 1:** Exploratory factor analysis of the different scales in the battery of questions

	Number of items	Number of factors	KMO	Bartlett’s test (*p*)	% variance explained	CFI	RMSEA
Risk factors	9	2	0.74	< 0.001	71.1	0.96	0.10
Signs and symptoms	9	2	0.69	< 0.001	58.8	0.97	0.08
Perception	6	1	0.65	< 0.001	46.7	0.94	0.15
Barriers	7	1	0.80	< 0.001	52.5	0.98	0.09

*KMO* Kaiser–Meyer–Olkin test, *CFI* comparative fit index, *RMSEA* root mean square error of approximation

**Table 2:** Correlation matrix between the different scales in the battery of questions

	Signs and symptoms (specific)	Signs and symptoms (non-specific)	Signs and symptoms	Perception	Barriers
Non-modifiable risk factors	0.056	0.364	0.313	0.261	− 0.104
Modifiable risk factors	0.054	0.340	0.293	0.415	− 0.091
Risk factors (total)	0.068	0.434	0.374	0.413	− 0.121
Signs and symptoms (specific)				0.163	− 0.079
Signs and symptoms (non-specific)				0.211	− 0.128
Signs and symptoms (total)				0.245	− 0.139
Perception					− 0.136

#### Descriptive statistics, reliability, and temporal stability of scores

Regarding the descriptive statistics of the items, the kurtosis of items three and four of dimension 1 of the signs and symptoms scale and the kurtosis of items 2 and 4 of dimension 2 of this subscale stand out in particular (Table 3). Reliability was adequate for all scales (Table 3). In addition, with the exception of the specific signs and symptoms scale, the temporal stability of the scores was shown to be adequate (Table 4).

**Table 3:** Descriptive statistics, factor weights, and reliability of the scales in the battery of questions

Subscale	Item	Mean	Standard deviation	Minimum	Maximum	Skewness	Kurtosis	Factor weight	α	ω
Non-modifiable risk factors	01 TH	0.47	0.5	0	1	0.12	− 1.99	0.48	0.85	0.86
	02 Menarche	0.12	0.32	0	1	2.37	3.61	0.79		
	03 Menopause	0.21	0.21	0	1	1.42	0.02	0.73		
	04 Infertile	0.27	0.27	0	1	1.02	− 0.96	0.76		
	05 Children < 30	0.21	0.21	0	1	1.46	0.13	0.86		
	Total	1.28	1.40	0	5	1.04	0.228	–		
Modifiable risk factors	01 Fats	0.71	0.71	0	1	− 0.91	− 1.18	0.84	0.92	0.92
	02 Overweight	0.72	0.72	0	1	− 0.98	− 1.05	0.99		
	03 Smoking	0.87	0.87	0	1	− 2.21	2.90	0.9		
	04 Wine	0.34	0.34	0	1	0.66	− 1.57	0.65		
	Total	2.64	1.31	0	4	− 0.78	0.23	–		
Risk factors (total)		3.92	2.21	0	9	0.15	− 0.45	–	0.88	0.88
Signs and symptoms (specific)	01 Swelling	0.92	0.28	0	1	− 3.04	7.25	0.68	0.74	0.74
	02 Breast pain	0.72	0.45	0	1	− 0.97	− 1.06	0.6		
	03 Lump in armpit	0.95	0.22	0	1	− 3.99	14.03	0.74		
	04 Lump in breast	0.95	0.21	0	1	− 4.30	16.55	0.54		
	Total	3.54	0.74	0	4	− 1.81	3.73	–		
Signs and symptoms (non-specific)	01 Discharge	0.83	0.38	0	1	− 1.73	1.00	0.64	0.84	0.84
	02 Changes	0.93	0.26	0	1	− 3.37	9.40	0.56		
	03 Tightness	0.47	0.5	0	1	0.10	− 2.00	0.59		
	04 Stretch marks	0.04	0.21	0	1	4.48	18.09	0.51		
	05 Dimple	0.53	0.5	0	1	− 0.14	1.99	1		
	Total	2.81	1.2	0	5	− 0.43	− 0.46	–		
Signs and symptoms (total)		6.34	1.53	0	9	− 0.74	0.72	–	0.81	0.81
Perception	1	4.38	0.73	1	5	− 1.03	0.83	0.67	0.77	0.77
	2	4.59	0.64	1	5	− 1.70	3.36	0.50		
	3	3.89	0.84	1	5	− 0.30	− 0.22	0.39		
	4	4.85	0.40	1	5	− 2.56	6.11	0.42		
	7	4.01	0.83	1	5	− 0.25	− 0.92	0.83		
	8	4.03	0.84	1	5	− 0.42	− 0.47	0.76		
	Total	25.74	2.75	6	30	− 0.35	− 0.25	–		
Barriers	1	1.93	1.03	1	5	1.02	0.41	0.69	0.85	0.85
	2	1.75	0.9	1	5	1.16	1.10	0.70		
	3	1.71	1.00	1	5	1.33	0.83	0.78		
	6	1.68	0.88	1	5	1.31	1.25	0.74		
	7	2.65	1.19	1	5	0.40	− 0.68	0.46		
	8	2.29	1.30	1	5	0.63	0.89	0.59		
	9	1.92	1.13	1	5	1.07	0.20	0.70		
	Total	13.92	4.91	7	35	0.80	0.31	–

**Table 4:** Temporal stability of the scores of the different scales in the battery of questions

	Intraclass correlation [95% CI]
Non-modifiable risk factors	0.937 [0.868; 0.970]
Modifiable risk factors	0.892 [0.772; 0.949]
Risk factors (total)	0.952 [0.900; 0.977]
Signs and symptoms (specific)	0.328 [-0.369; 0.675]
Signs and symptoms (non-specific)	0.718 [0.415; 0.865]
Signs and symptoms (total)	0.654 [0.287; 0.834]
Perception	0.885 [0.760; 0.945]
Barriers	0.866 [0.720; 0.936]

#### Evidence of validity in relation to other variables

Both modifiable and non-modifiable risk factor scales showed a correlation with the breast cancer knowledge questionnaire of 0.341 [95% CI = 0.271; 0.408] and 0.434 [95% CI = 0.369; 0.495], respectively. In turn, the breast cancer knowledge questionnaire showed a correlation of 0.394 [95% CI = 0.326; 0.457] with the non-specific signs and symptoms scale versus a correlation of 0.131 [95% CI = 0.054; 0.206] with the specific signs and symptoms scale. Finally, the perceived risk scale showed a correlation of 0.320 [95% CI = 0.248; 0.388] with the breast cancer knowledge questionnaire, whereas the barriers scale showed a correlation of − 0.230 [95% CI =  − 0.155; − 0.303].

#### Evidence of validity with regard to discriminatory power

Differences in modifiable risk factors are apparent, with women with a family history of cancer scoring significantly higher. Differences can also be identified on the barriers scale, with women with no history of breast cancer scoring significantly higher (Table 5). Finally, it is also important to note that women with higher levels of perceived risk exhibited lower knowledge of barriers and greater knowledge of signs and symptoms (Table 6). Women with a university education scored higher (*p* < 0.001) on non-modifiable risk factors (*d* = 0.43), modifiable risk factors (*d* = 0.36), and all factors (*d* = 0.49) than women without university education. Women with a university education also displayed greater knowledge of non-specific signs and symptoms (*p* = 0.014; *d* = 0.22) and all signs and symptoms (*p* = 0.005; *d* = 0.95), but showed no difference in specific signs and symptoms (*p* = 0.094; *d* = 0.15). Finally, no differences were found regarding perceptions (*p* = 0.313; *d* = 0.09) or barriers (*p* = 0.099; *d* = 0.14) based on their level of education.

**Table 5:** Differences between the scales in the battery of questions depending on the presence or absence of a history of cancer

	Family history of cancer				Family history of breast cancer			
	*M* No	*M* Yes	t (*p*)	*d*	*M* No	*M* Yes	t (*p*)	*d*
Non-modifiable risk factors	1.25	1.3	− 0.37 (0.709)	0.04	1.24	1.34	− 0.88 (0.379)	0.08
Modifiable risk factors	2.36	2.73	− 2.72 (0.007)	0.29	2.61	2.63	− 0.16 (0.877)	0.02
Risk factors (total)	3.61	4.03	− 1.84 (0.068)	0.19	3.85	3.97	− 0.65 (0.518)	0.06
Signs and symptoms (specific)	3.48	3.56	− 1.06 (0.288)	0.1	3.55	3.5	0.84 (0.402)	0.08
Signs and symptoms (non-specific)	2.61	2.86	− 2.22 (0.027)	0.21	2.72	2.83	− 0.98 (0.328)	0.09
Signs and symptoms (total)	6.09	6.42	− 2.25 (0.025)	0.22	6.28	6.33	− 0.36 (0.717)	0.03
Perception	25.52	25.81	− 1.11 (0.266)	0.11	25.8	25.56	1.01 (0.313)	0.09
Barriers	13.92	13.96	− 0.86 (0.931)	0.01	14.49	12.76	4.22 (< 0.001)	0.35

*M* mean, *t* testing statistic, *p* level of statistical significance, *d* effect size

**Table 6:** Differences between the scales in the battery of questions depending on the degree of perceived risk and feelings of concern

	Perceived risk							Feelings of concern						
	*M* N	*M* L	*M* Mo	*M* H	F (*p*)	*d*	Post hoc	*M* N	*M* L	*M* Mo	*M* H	F (*p*)	*d*	Post hoc
Modifiable RFs	0.88	1.21	1.32	1.46	1.09 (0.353)	0.09		1.33	1.26	1.2	0.94	0.85 (0.469)	0.07	
Non-modifiable RFs	1.94	2.55	2.72	2.82	2.75 (0.042)	0.22		2.52	2.77	3.05	2.48	3.84 (0.010)	0.31	a with c
RFs (total)	2.81	3.76	4.04	4.28	2.70 (0.045)	0.22		3.85	4.04	4.25	3.42	1.29 (0.278)	0.11	
SS (specific)	3.13	3.59	3.51	3.53	2.19 (0.089)	0.17		3.55	3.53	3.52	3.39	0.49 (0.691)	0.04	
SS (non-specific)	2.06	2.77	2.87	2.88	2.46 (0.062)	0.2		2.75	2.82	3.08	2.87	1.43 (0.232)	0.12	
SS (total)	5.19	6.36	6.38	6.41	3.20 (0.023)	0.26	a with b a with c a with d	6.3	6.35	6.6	6.26	0.74 (0.527)	0.06	
P	24	25.9	25.8	25.4	2.76 (0.041)	0.22		25.8	25.6	26.1	25.1	1.22 (0.301)	0.1	
B	18.4	14	13.8	13.2	4.43 (0.004)	0.36	a with b a with c a with d	14.1	13.7	13.4	14.1	0.50 (0.683)	0.05	

*RFs* risk factors, *SS* signs and symptoms, *P* perception, *B* barriers, *N* none, *L* low, *Mo* moderate, *H* high, *M* mean, *F* testing statistic, *d* effect size, *p* level of statistical significance

## Discussion

The MARA questionnaire for assessing knowledge of breast cancer, perceived risk of developing breast cancer, and perceived barriers regarding breast cancer prevention has been shown to have adequate psychometric properties for use in Spanish-speaking women. To the best of our knowledge, there is no tool available in Spanish that has the same characteristics as the MARA questionnaire. However, its content, structure, and psychometric properties are similar to those observed in questionnaires developed in other languages [8, 9, 11].

The MARA questionnaire consists of four scales: risk factors, signs and symptoms, perceived risks, and perceived barriers. The risk factors scale displayed a two-dimensional structure, with two subscales: modifiable risk factors and non-modifiable risk factors. The signs and symptoms scale also exhibited a two-dimensional structure consisting of specific signs and symptoms—associated with breast cancer by the general population—and non-specific signs and symptoms—associated with other breast conditions by the general population. Both scales (risk factors and signs and symptoms) showed a strong correlation between their subscales, allowing a total score to be obtained. Both the perceived risks and the barriers scales exhibited an essentially one-dimensional structure. Along with the fact that all the scales showed strong correlations between one another, this demonstrates that the scales constitute a significant body of knowledge on breast cancer.

Internal consistency was adequate for all scales, as shown by both Cronbach’s alpha and McDonald’s omega coefficients, ranging from 0.77 to 0.92. The scales also show adequate temporal stability in general [20]. In terms of evidence of validity in relation to other variables, the battery of questions in the MARA showed evidence of convergent validity [21], given that all the scales that it comprises showed strong correlations with the breast cancer knowledge questionnaire [13]. Having adequate psychometric properties is essential for the safe use of this type of tool, as suggested by other authors [8, 9].

With respect to the discriminatory power of the battery of questions, differences in the risk factors scale based on the presence or absence of a family history of cancer stand out, with women with a family history of cancer scoring significantly higher. In addition, when looking at the barriers scale, women with no history of breast cancer show significantly higher scores. These results are consistent with those observed in previous studies. In addition, several authors suggest that having loved ones with a history of cancer acts as a facilitator for the development of preventive attitudes [22] and behaviors [23].

The MARA questionnaire has been shown to be effective in assessing knowledge of risk factors related to breast cancer, signs and symptoms of breast cancer, perceived individual risk of developing breast cancer, ability to engage in preventive behaviors, and barriers to breast cancer prevention. This information can help healthcare workers to meet women’s needs in relation to breast cancer prevention.

On a personal level, possessing knowledge increases intentions to adopt healthy behaviors [24]. Specifically, in the case of breast cancer, it acts as a mediating factor in predicting breast cancer screening practice [5, 25]. Additionally, as the HBM suggests, an absence of knowledge directly affects perception [2].

Failure to perceive the existence of a health problem may lead to a failure to take action to prevent or address said problem. When women are aware of the risk factors for breast cancer, they are better placed to perceive their actual risk of developing the disease [26]. This can be used in clinical practice to implement individualized prevention and screening strategies [27].

In addition, identifying needs relating to the dimensions covered by the MARA allows these needs to be addressed and met, which can help women to take an active role not only in preventing the condition, but also in detecting it promptly. It should be borne in mind that prevention plans in Spain are standardized and their inclusion criteria are essentially based on women’s age and individual and family history of breast cancer. For example, women and immediate relatives of women who have not had breast cancer are included in prevention programs from the age of 50 onward, which does not appear reasonable given the multiple causes associated with this type of cancer. This is demonstrated in a study by Mukama et al. [28], which suggests a need to establish specific screening plans for these women and not to select exclusively on the basis of personal or family history. In the same vein, other authors have stated that public health initiatives should prioritize breast cancer prevention and early detection among women, paying special attention to women with a lack of knowledge, perception, and awareness of this type of cancer [29].

One limitation of our study is that the sample may not be representative. Culture and society determine behavior, so the items of the MARA should be reviewed before use in other Spanish-speaking regions, such as Hispanic America.

However, our study also has a number of strengths. Confirming the psychometric properties of the MARA questionnaire has important clinical implications. Firstly, the MARA questionnaire can be used in healthcare and gynecological facilities to assess Spanish women’s knowledge, risks, perceptions, and barriers regarding breast cancer. Secondly, the independent measurement of these aspects facilitates the identification of specific individual needs so that professionals can better address them and take steps to develop effective interventions. Finally, the results of such an assessment may be used to inform educational and counseling interventions for addressing women’s misconceptions and improving their knowledge about breast cancer.

## Conclusion

The MARA questionnaire has adequate internal consistency and an adequate factor structure. It therefore represents a valid, reliable tool for assessing Spanish women’s knowledge, risks, perceptions, and barriers regarding breast cancer.

## References

[1] Schüz J, Espina C, Wild CP (2019). Primary prevention: a need for concerted action. Mol Oncol.

[2] Coleman MT, Pasternak RH (2012). Effective strategies for behavior change. Prim Care.

[3] Michie S, van Stralen MM, West R (2011). The behaviour change wheel: a new method for characterising and designing behaviour change interventions. Implement Sci.

[4] Marzo-Castillejo M, Vela-Vallespín C, Bellas-Beceiro B (2018). Recomendaciones de prevención del cancer. Actualización PAPPS 2018. Aten Prim.

[5] Rainey L, van der Waal D, Wengström Y, Jervaeus A, Broeders M (2018). Women's perceptions of the adoption of personalised risk-based breast cancer screening and primary prevention: a systematic review. Acta Oncol.

[6] Ozanne E, Karliner LS, Tice JA (2019). An intervention tool to increase patient-physician discussion of lifestyle risk factors for breast cancer. J Womens Health.

[7] Sanz-Barbero B, Prieto-Flores ME, Otero-García L, Abt-Sacks A, Bernal M, Cambas N (2014). Perception of risk factors for cancer in the Spanish population. Gac Sanit.

[8] Linsell L, Forbes LJ, Burgess C, Kapari M, Thurnham A, Ramirez AJ (2010). Validation of a measurement tool to assess awareness of breast cancer. Eur J Cancer.

[9] Solikhah S, Promthet S, Rakkapao N, Hurst CP (2017). Validation of an Indonesian version of the breast cancer awareness scale (BCAS-I). Asian Pac J Cancer Prev.

[10] Córdoba García R, Camarelles Guillem F, Muñoz Seco E (2018). Recomendaciones sobre el estilo de vida. Actualización PAPPS 2018. Aten Prim.

[11] Rakkapao N, Promthet S, Moore MA, Hurst CP (2016). Development of a breast cancer awareness scale for Thai women: moving towards a validated measure. Asian Pac J Cancer Prev.

[12] World Health Organization (2016) Process of translation and adaptation of instruments. http://www.who.int/substance_abuse/research_tools/translation/en/. Accesed 24 October 2020

[13] Seven M, Bağcivan G, Akyuz A, Bölükbaş F (2018). Women with family history of breast cancer: how much are they aware of their risk?. J Cancer Educ.

[14] Elosua Oliden P, Zumbo BD (2008). Coefficients of feasibility for ordinal response scales. Psicothema.

[15] McDonald RP (1999). Test theory: a unified treatment.

[16] Timmerman ME, Lorenzo-Seva U (2011). Dimensionality assessment of ordered polytomous items with parallel analysis. Psychol Methods.

[17] Hu LT, Bentler PM (1999). Cutoff criteria for fit indexes in covariance structure analysis: conventional criteria versus new alternatives. Struct Equ Model.

[18] Cohen J (1988). Statistical power analysis for the behavioral sciences.

[19] Ferrando PJ, Lorenzo-Seva U (2017). Program FACTOR at 10: origins, development and future directions. Psicothema.

[20] Muñiz J, Fonseca-Pedrero E (2019). Ten steps for test development. Psicothema.

[21] Evers A, Muñiz J, Hagemeister C (2013). Assessing the quality of tests: revision of the EFPA review model. Psicothema.

[22] Haug U, Riedel O, Cholmakow-Bodechtel C, Olsson L (2018). First-degree relatives of cancer patients: a target group for primary prevention? A cross-sectional study. Br J Cancer.

[23] Bostean G, Crespi CM, McCarthy WJ (2013). Associations among family history of cancer, cancer screening and lifestyle behaviors: a population-based study. Cancer Causes Control.

[24] Frieden TR (2010). A framework for public health action: the health impact pyramid. Am J Public Health.

[25] Firouzbakht M, Hajian-Tilaki K, Bakhtiari A (2020). Comparison of competitive cognitive models in explanation of women breast cancer screening behaviours using structural equation modelling: health belief model and theory of reasoned action. Eur J Cancer Care.

[26] Chung C, Lee SJ (2013). Estimated risks and optimistic self-perception of breast cancer risk in Korean women. Appl Nurs Res.

[27] Lippey J, Keogh LA, Mann GB, Campbell IG, Forrest LE (2019). "A natural progression": Australian women's attitudes about an individualized breast screening model. Cancer prev res.

[28] Mukama T, Fallah M, Tian Y (2020). Risk-tailored starting age of breast cancer screening based on women's reproductive profile: a nationwide cohort study. Eur J Cancer.

[29] Yeung M, Chan E, Wong S, Yip B, Cheung PS (2019). Hong Kong female's breast cancer awareness measure: cross-sectional survey. World J Clin Oncol.

